# Even Considering the Existing High Technology, Do Not Forget That the Old Stethoscope Is Still a Useful Tool for the Heart Team

**DOI:** 10.21470/1678-9741-2018-0604

**Published:** 2018

**Authors:** Paulo Roberto B. Evora, André Schmidt, Domingo M. Braile

**Affiliations:** 1 Editor-in-Chief Interim - BJCVS Faculdade de Medicina de Ribeirão Preto da Universidade de São Paulo (FMRP-USP), Ribeirão Preto, SP, Brazil; 2 Faculdade de Medicina de Ribeirão Preto da Universidade de São Paulo (FMRP-USP), Ribeirão Preto, SP, Brazil; 3 Editor-in-Chief - BJCVS Faculdade de Medicina de São José do Rio Preto (FAMERP), São José do Rio Preto, SP, Brazil and Universidade de Campinas (UNICAMP), Campinas, SP, Brazil.

## BJCVS Highlight

*"The most important part of an art is to be able to observe properly"*

René-Théophile-Hyacinthe Laennec

In 2016, the stethoscope completed 200 years since its first monaural description by Rene Laennec, in 1896. To the best of our knowledge, no national publication has highlighted this major event. The invention of the familiar "binaural" stethoscope is credited to the Irish physician Arthur Leared, in 1851. The first binaural stethoscope commercially produced was developed by George Camman, in 1852, in New York, USA.

After these brief historical vignettes, Harbison^[^^1^^]^ emphasizes that since 1816 a number of technologies have been developed to challenge the stethoscope: X-rays in the 1890s, diagnostic ultrasound in the 1950s and computed tomography in the 1970s, but none have adequately replaced it. Nowadays, its real value has been argued in some editorials ^[^^2^^,^^3^^]^, and passionate defenses were published ^[^^4^^]^. However, it is important not to underestimate it as a diagnostic tool where complex technology is not available. Besides detecting bronchospasm, the stethoscope may still overcome an echocardiogram in cases of diastolic heart failure and may be an adjuvant tool in the diagnosis of acute respiratory distress syndrome (ARDS). Indeed, requesting a chest computed tomography (CT) or echocardiogram from a radiologist or cardiologist without being able to describe the auscultatory findings probably will trigger a curt response ^[^^1^^]^. Remember that the stethoscope should be a good idea against the diagnosis of "moderate injury" described on imaging, often avoiding cardiac valve prosthesis surgery.

Almost 50 years ago, Ashbaugh et al. ^[^^5^^]^ ﬁrst used the term ''acute respiratory distress in adults'' to describe 12 patients with respiratory failure. The syndrome hallmarks associated with respiratory distress were hypoxemia refractory to supplemental oxygen, diffuse radiographic opacities, and histological evidence of diffuse alveolar damage in most, but not all, fatal cases. Later, Petty and Ashbaugh ^[^^6^^]^ created the final term ARDS. Considering the strong historical association between ARDS and the Vietnam War and the sophistication of current imaging methods ^[^^7^^,^^8^^]^, we remembered the 1970s, when the stethoscope and a simple chest X-ray were our weapons. Interstitial pulmonary edema occurring in association with non-cardiac disease (*e.g*., sepsis, aspiration or shock) is secondary to an increase in the permeability of the pulmonary microvasculature and is the pathophysiological basis of ARDS. It is said that ARDS is a "barrier problem" (alveolar-capillary barrier). The radiological image, although often exuberant, is incompatible with the findings on pulmonary auscultation and the amount of pulmonary secretions present. Here, pulmonary auscultation is poor in findings, and this is the hallmark for the diagnosis of this condition. The diagnosis of ARDS is made by cardiologists in intensive care units (ICU), usually in postoperative patients, and includes respiratory distress and progressive arterial desaturation with a decrease in PaCO_2_. In the 1970s, during ICU rounds, the ARDS chest X-ray films (especially ARDS class III) invariably had the diagnosis of "extensive bilateral pneumonia". We must have repeated hundreds of times that ARDS is a "barrier" problem, and the radiological image was incompatible with the findings of auscultation and pulmonary secretions.

In conclusion, we were living in an era of advanced imaging techniques, but excellent clinical observation (especially the time course of symptom development) and the use of this "ancient" 200 year-old tool may rule out many confounding diagnoses, indicate the appropriate treatment, or dictate advanced investigation.

## Articles in this Issue

This issue of Brazilian Journal of Cardiovascular Surgery (BJCVS) presents a blind peer-reviewed selection of 16 papers that will surely please our readers. The vast majority is related to various perioperative problems. We selected 15 articles in order of acceptance (10 original articles, 2 review articles, and 3 elected case reports) and 1 editorial addressed to the fundamental concepts to Biostatistics.

**Paulo Roberto B. Évora** ^1^Editor-in-Chief Interim - BJCVS Faculdade de Medicina de
Ribeirão Preto da Universidade de São Paulo (FMRP-USP),
Ribeirão Preto, SP, Brazil**André Schmidt** ^2^Faculdade de Medicina de Ribeirão Preto da Universidade
de São Paulo (FMRP-USP), Ribeirão Preto, SP, Brazil**Domingo M. Braile** ^3^Editor-in-Chief - BJCVS Faculdade de Medicina de São
José do Rio Preto (FAMERP), São José do Rio Preto, SP,
Brazil and Universidade de Campinas (UNICAMP), Campinas, SP, Brazil.
